# Healthy Aging in Colombia 2018 and Its Variation in Relation to Social Conditions

**DOI:** 10.3390/ijerph21091244

**Published:** 2024-09-20

**Authors:** Yesika Natali Fernández-Ortiz

**Affiliations:** Pontificia Universidad Javeriana, Institute of Public Health, Bogotá 110231, Colombia; fernandez.y@javeriana.edu.co; Tel.: +57-3208320-2235

**Keywords:** older adults, healthy aging, disability-free life expectancy, inequalities, Colombia

## Abstract

The population aging in the region is occurring under scenarios of inequality, raising concerns about how the increase in life expectancy is experienced and what factors affect the quality of life of older adults. This research quantified the differentials of healthy aging in Colombia in 2018 and its association with social indicators through a cross-sectional, descriptive, and correlational observational study. Healthy aging was quantified using the Disability-Free Life Expectancy (DFLE) indicator and later correlated with social indicators and subjected to a Multiple Factor Analysis (MFA). The results showed a healthy life expectancy of 71.5 years for women and 66.9 years for men, with a disability expectancy of 8.3 and 6.4 years, respectively. Negative associations emerged with health problems, disability, lack of medical care, illiteracy, school absenteeism, and poverty, while higher education levels and retirement showed positive associations. The factor analysis by area of residence highlighted urban areas as conducive to healthy aging. In conclusion, the accelerated aging of the Colombian population faces health disparities that policies must address by improving education, economic security, and health services, especially for women and rural areas.

## 1. Introduction

The demographic transition, as demographer Frank Notestein describes, refers to the shift from high to low levels of fertility and mortality. This process has resulted in a notable increase in the aging population, with a proportional rise in the number of individuals older than 60 years old compared to those under 15 years of age. This trend, driven by medical advancements that have extended Life Expectancy (LE) in both developed and developing countries, is expected to become a global reality by 2050 [1].

However, some experts warn that gains in life expectancy are at risk due to the increasing prevalence of disabling chronic diseases and emerging pandemics [2]. This is particularly concerning for the elderly population, where these conditions lead to higher levels of disability, dependence, and a decline in quality of life, with significant impacts on both individuals and their familial and social environments [1].

In this context, it is essential to monitor not only life expectancy but also the quality of those additional years by using indicators such as Disability-Free Life Expectancy (DFLE) [3] (Gispert et al., 2007). This indicator allows assessment in order to check if the population is experiencing an expansion of morbidity—as suggested by Gruenberg [4], additional years of life show increased illness and disability—or reduced morbidity, as Fries [5] proposes, where additional years are predominantly healthy.

For a time, researchers struggled to calculate DFLE in Latin America due to the scarcity of longitudinal studies on disability with representative statistical samples [6] (However, in 2001, the UN Disability Statistics Group recommended that censuses and surveys include standardized questions on disability, facilitating the analysis of this condition, which enabled the calculation of indicators like DFLE for international comparisons [6]).

This advancement allowed for the exploration of the relationship between DFLE and social variables, demonstrating that factors such as education, income, and geographic location can influence the quality of life in old age. Recent research by Brønnum-Hansen et al. [7] and Van Oyen et al. [8] has revealed the impact of educational levels on DFLE, while studies by Wagg et al. [9], Wu et al. [10], and Brønnum-Hansen et al. [11] have highlighted the influence of higher income. Additionally, other works have delved into geographic variations and their relationship with gender, education, unemployment rates, poverty, and access to healthcare services [12,13,14,15,16,17,18,19,20]. These studies underscore the importance of social factors in healthy life expectancy and justify the need for differentiated approaches based on social and economic contexts.

In Latin America, and particularly in Colombia, population aging presents specific challenges due to the profound social and economic inequalities that characterize the region. According to data from the 2018 National Population and Housing Census, the percentage of the population aged 65 and older in Colombia increased from 6.3% in 2005 to 9.1% in 2018. During this period, the Aging Index rose from 16.7 to 58.7 older adults per 100 individuals under 15 years of age, reflecting the rapid aging of the population [21].

This process has been accompanied by an increase in the prevalence of disability, which rose from 33% in 2005 to 41% in 2018 [22]. Additionally, the Economic Commission for Latin America and the Caribbean (ECLAC) reported that Disability Expectancy (DE) in Colombia increased from 5.2 to 5.9 years between 2000 and 2015, which indicates that the country experienced an expansion of morbidity [23]. Despite this information, no one has updated the latter indicator since 2018, so we do not know whether the years lived with disability or DE have increased in recent years or how their distribution and variation within the country relate to socioeconomic and geographic variables [24]. Therefore, this study hypothesizes that disability among the elderly population reduces healthy life expectancy and that it is possible to identify significant health disparities when comparing these outcomes with socioeconomic characteristics.

To test this hypothesis, the study aimed to quantify the differentials in healthy aging within Colombia through DFLE and explore its possible association with social indicators for 2018. These measurements offered insights into the factors influencing the attainment of healthy life years by sex and area of residence.

The relevance of this research lies in an estimate of the healthy life years that the Colombian population achieved in 2018, confirming the expansion of morbidity through DE and demonstrating the existence of social inequalities that affect development opportunities and healthy aging. These findings are essential for policymakers to strengthen responses to the challenges of aging, reduce disparities, and promote healthy aging in Colombia within the context of growing demographic and social challenges.

## 2. Materials and Methods

### 2.1. Design and Data Source

This observational, cross-sectional, and correlational study utilized data from the 2018 National Population and Housing Census (NPHC) and vital statistics, both obtained from the National Administrative Department of Statistics [21] of Colombia.

The NPHC questionnaire collected detailed data on the demographic, social, and economic characteristics of the population [25]; this gave information regarding the population over 60 years old by department, age, sex, area of residence (urban/rural), health outcomes (questions 42–45), education (questions 50 and 51), and socioeconomic conditions (question 52).

The REDATAM (Recovery of Data for Small Areas by Microcomputer) platform of the NPHC 2018 provided data on the Multidimensional Poverty Index (MPI); in the same way, technical publications by sex, area, and department [26,27] gave information about life tables for Colombia 2018. Additionally, DANE [28] contributed vital statistics on death records.

It is important to note that the disaggregation by areas of residence was performed to differentiate urban areas (municipal capitals), which have better access to services and socioeconomic conditions, from rural areas (small towns and dispersed rural areas), which face greater barriers in education, health, and economic development.

Regarding the analysis of departments, it could follow the geographic region patterns established by the Agustín Codazzi Geographic Institute (IGAC), such as the Andean, Caribbean, Insular, Pacific, Orinoquía, and Amazon regions [29]. However, this study examines the results according to the classification of areas of residence. This approach allows for a more accurate understanding of how living conditions and available services impact DFLE in Colombia, differentiating between urban and rural areas.

### 2.2. Data Processing

The study transformed the variables of interest into indicators according to sex and area (urban and rural). Table 1 below details the operationalization of the variables for their use as indicators.

This research calculated DFLE using the method that Sullivan proposed in the 1970s, which integrates life tables with disability prevalence rates to estimate the years lived with and without disability, considering the latter as healthy life years or DFLE [30,31,32]. This method combines information on mortality and morbidity to provide a comprehensive view of the population’s health.

$$DFLE_{x}=\frac{\sum_{i=x}^{90}\left((1-t_{i})L_{i}\right)}{l_{x}}$$

The calculation of DFLE used life tables published by DANE from the CNPV 2018. It also calculated the prevalence of disability based on affirmative responses to Census question No. 44 (In your daily life, do you have difficulties performing activities such as: hearing, speaking, seeing, moving your body, walking, grasping objects with your hands, understanding, learning or remembering, eating or dressing by yourself, and interacting with others?). The analysis then disaggregated this information by age group, sex, and area and then divided it by the population.

To further explore healthy life expectancy and identify the number of years a population with disabilities or poor health is likely to live, the calculation of DE involved subtracting DFLE from LE (DFLE − LE).

Pearson bivariate correlations and Multiple Factor Analysis (MFA) identified associations between DFLE and social indicators. Pearson correlations assessed the strength and direction of linear relationships between variables, while MFA allowed for the simultaneous examination of multiple variables, identifying patterns and relationships within the data [33].

Before performing the MFA, statistical methods tested the assumptions of linearity and normality of the variables. Visual inspection of scatter plots confirmed linearity, and the Shapiro–Wilk test assessed normality. The results indicated that the variables met the necessary criteria for the application of MFA, ensuring the validity of the analysis.

Table 2 provides a summary of the variables along with the Shapiro–Wilk test’s *W* statistic and *p*-value. Of the total variables (68 in total), 96% had a *W* statistic value close to 1, and 65% had a *p*-value greater than 0.05, indicating that the variables follow a normal distribution, which allows for the acceptance of the null hypothesis (H_0_). The study included all variables, as MFA relies on the prior normalization of data through Principal Component Analysis (PCA), enabling the handling and combining of different sets of variables for joint analysis [34].

The statistical analysis and result visualization used R version 4.1.2, along with the FactoMineR and Factoextra packages.

For the MFA, the analysis grouped the indicators into seven categories by area and sex: G1 (Education), G2 (Socioeconomic), G3 (Perceived Morbidity), G4 (Mortality), G5 (Health Care), G6 (DFLE at Birth), and G7 (DFLE at Age 60). The individuals in the analysis were the departments; the study assigned a label to each indicator in order to differentiate them by area and sex. The letter “C” represents urban areas, and “R” represents rural areas (Table 3). To differentiate by sex, the letter ‘H’ represents men and ‘M’ represents women.

## 3. Results

According to the 2018 Census, 75.67% (4,704,419) of the elderly population resided in urban areas, while 24.33% (1,512,429) lived in rural areas. Women were more concentrated in urban areas (43.16%), highlighting the feminization of aging in cities, whereas men had a slightly higher representation in rural areas (12.86%) [26]. These data emphasize the unequal distribution of the elderly population by area of residence and gender, underscoring the need to analyze results by area of residence to provide insights that guide the development of differentiated policies tailored to the specific needs of each group.

### 3.1. Disability-Free Life Expectancy and Disability Expectancy at Birth

The 2018 Census also revealed that life expectancy at birth in Colombia was higher for women (79.8 years) compared to men (73.3 years); this distribution remained consistent across all geographic areas. Additionally, LE was higher in urban areas compared to rural areas. For men, DFLE dropped to 66.9 years, while for women, it reached 71.5 years, resulting in a DE of 8.3 years for women and 6.4 years for men. Rural areas showed a lower DFLE compared to urban areas, particularly among women (Table 4).

Departments with the highest and lowest DFLE differed by 16.4 years for men and 17.1 years for women. The geographic analysis revealed that departments with better socioeconomic conditions, which are generally urban and economic centers, have higher LE and DFLE. In contrast, more rural departments and those further from economic centers had lower values; this highlights the importance of addressing regional inequalities in health and other social services.

Pearson bivariate correlations showed significant associations between DFLE and various social indicators. The MFA identified patterns and relationships within the data and showed that higher education levels, better socioeconomic conditions, and improved healthcare services positively influence higher DFLE. Conversely, higher morbidity and mortality negatively affected DFLE. These findings emphasize the importance of targeted policies to improve education, economic security, and health services, especially in rural areas and among women (Table 5).

### 3.2. Life Expectancy and Disability Expectancy at Age 60

At age 60, men had a life expectancy of 21.1 years, while women had 24.1 years. DFLE decreased to 16.4 years for men and 18.0 years for women. In rural areas, these values declined further due to higher rates of disability and mortality.

Regarding DE, men experienced 4.6 fewer healthy years compared to women, who had 6.1 years, indicating higher levels of disability. This trend affected rural areas more strongly (Table 6).

### 3.3. Bivariate Correlation of DFLE at Birth with Social Variables

The bivariate correlation analysis revealed interesting patterns. DFLE negatively correlated with perceived morbidity, disability (especially in women), and the lack of health service provision.

In terms of education, DFLE showed a negative relationship with illiteracy, school absenteeism, and the absence of any level of schooling, particularly among women. Conversely, there was a positive association between DFLE and higher educational attainment.

Socioeconomic conditions indicated a negative correlation between DFLE and the MPI, while living off retirement income showed a positive relationship (Table 7).

### 3.4. Multiple Factor Analysis (MFA)

The MFA helped identify groups of indicators that differently affect healthy aging according to the area of residence.

### 3.5. Multiple Associations in Urban Areas

In urban areas, the groups of DFLE at birth, DFLE at age 60, and education showed a high correlation, which partly explains why these areas have a better life expectancy. Additionally, the education group strongly correlated with socioeconomic indicators (0.74), while perceived morbidity related to DFLE at birth (0.48) and DFLE at age 60 (0.53), showing a strong representation in the common factor. Mortality and healthcare showed lower correlations and had a smaller representation (Table 8).

### 3.6. Distribution and Contribution of Indicators in Urban Area

**Figure 1:**
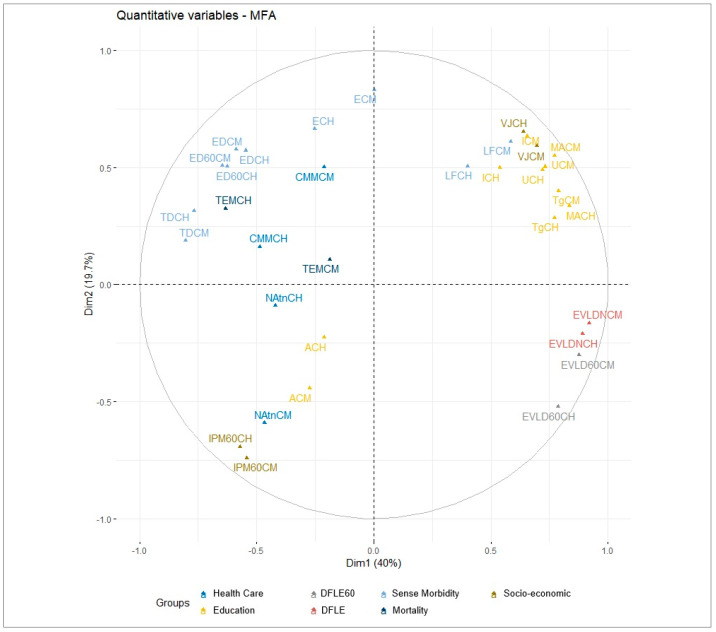
Variable MFA in urban area, Colombia 2018. Note: Own elaboration.

DFLE at birth, DFLE at age 60, living off retirement, and educational indicators maintained a high distribution and contribution by sex, positively correlating with dimensions 1 and 2. This suggests that high and positive values promote healthy aging. In contrast, disability, mortality, MPI, and lack of healthcare, by showing negative correlations with these dimensions, counteract healthy aging in urban areas (Figure 1).

**Figure 2:**
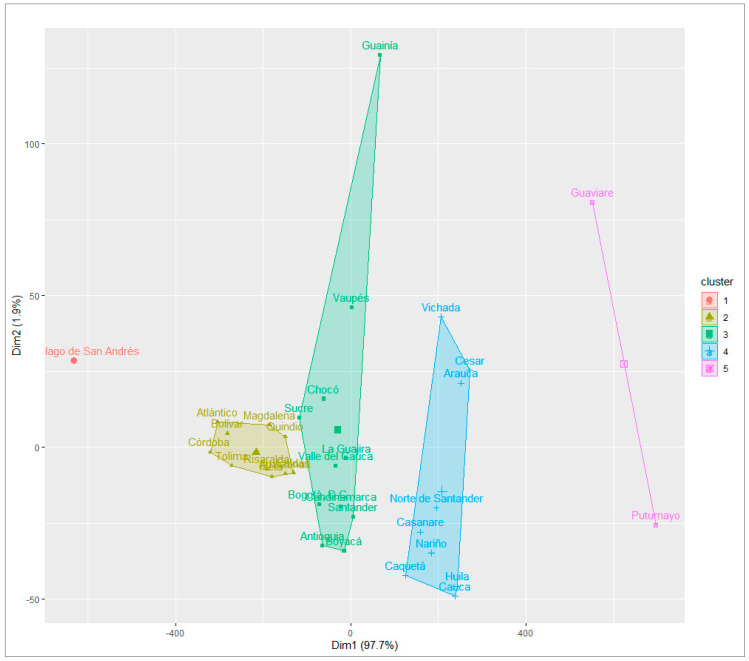
Cluster departments according to multiple associations of indicators of urban areas, Colombia 2018. Note: Own elaboration.

The distribution of departments showed a clear division: clusters 5 and 4 face unfavorable social conditions, high morbidity, disability, and mortality rates, and low levels of retirement, schooling, DFLE at birth, and DFLE at age 60. Cluster 3, with diverse social conditions, faces urban challenges due to migration and the saturation of health services. Clusters 2 and 1, with better health indicators and social conditions, exhibited high values of DFLE at birth and DFLE at age 60 and low levels of disability and mortality. It is noteworthy that cluster 1 includes only the department of the Archipelago of San Andrés and Providencia due to census omissions [35] (Figure 2).

### 3.7. Multiple Associations in Rural Areas

In rural areas, the education, socioeconomic status, perceived morbidity, and DFLE groups at birth showed higher correlations and good representation of the common factor. Among these groups, education and socioeconomic indicators strongly correlated (0.81), and perceived morbidity with DFLE at age 60 generated a robust correlation (0.52) (Table 9).

### 3.8. Distribution and Contribution of Indicators in Rural Areas

Unlike the distribution of indicators in urban areas, the indicators that favor healthy aging in rural areas appear on the negative side of dimension 1, while those that do not favor it correlate positively.

High values of the MPI, illiteracy, lack of healthcare, and perceived morbidity reduce DFLE at birth. Similarly, high disability and mortality rates lead to lower DFLE at age 60 and fewer retirement benefits.

**Figure 3:**
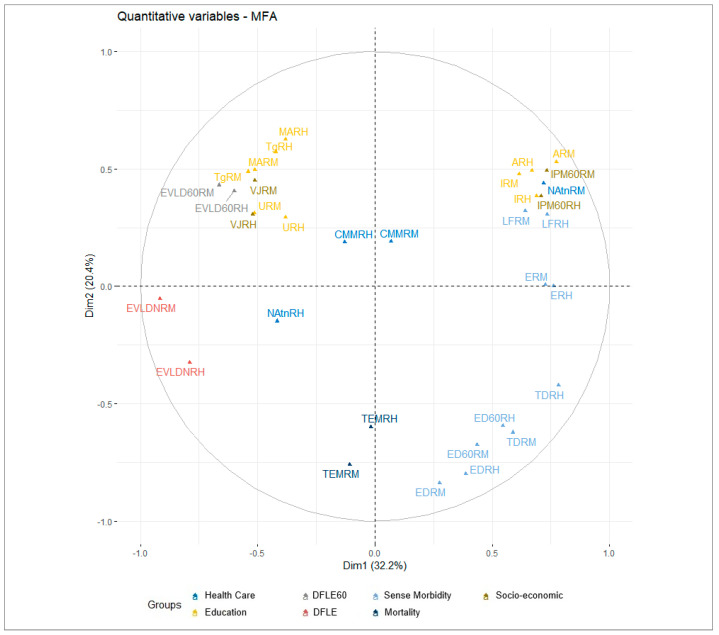
Variable MFA in populated rural areas, Colombia 2018. Note: Own elaboration.

For women, DFLE at age 60 is particularly low due to high mortality and disability rates; this hinders the achievement of healthy aging (Figure 3).

The distribution of departments in this area formed six clusters. Clusters 6 and 5 grouped departments with poor social conditions, high levels of precariousness in healthcare and education, high disability rates, and low DFLE at birth and age 60.

Clusters 4 and 3 included departments with varied social conditions. Those with worse conditions also lacked adequate healthcare, had high MPI, illiteracy, a higher disease burden, low DFLE at age 60, and school absenteeism. Departments with better conditions had higher levels of schooling, retirement benefits, and DFLE at birth.

**Figure 4:**
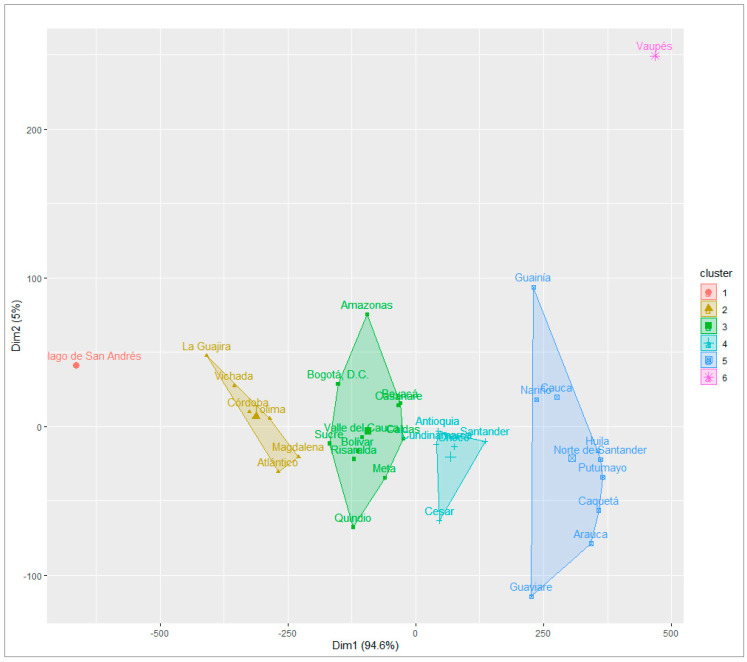
Departmental clusters according to multiple associations of indicators of the rural area, Colombia 2018. Note: Own elaboration.

Clusters 2 and 1 had the best indicators, favoring the health and social conditions of the elderly population. These included low mortality and disability rates and better educational indicators and DFLE for women (Figure 4).

The distribution of departments by clusters revealed a disassociation from traditional geographic regions, highlighting that the area of residence primarily influenced health disparities.

For example, rural areas in the Andean region departments (Cundinamarca, Boyacá, Antioquia, Santander) exhibit worse outcomes compared to urban areas despite being part of the same geographic region. This analysis underscores the need for differentiated public policies that address the specific disparities of each area of residence, focusing on improving access to essential services in rural areas to reduce health inequalities.

Finally, these results support the initial hypothesis that disability among the elderly population reduces healthy life expectancy. Additionally, analyzing socioeconomic characteristics reveals clear health disparities. Specifically, the prevalence of disability is higher among women and in rural areas, underscoring the importance of factors such as access to healthcare services, educational attainment, and financial security in the quality of life of older adults.

## 4. Discussion

The results of this study revealed significant differences in the distribution of DFLE and DE in Colombia in 2018, highlighting the socioeconomic and geographic inequalities that affect the quality of life of older adults. This research offers a novel contribution by providing updated estimates of DFLE for Colombia, emphasizing the expansion of morbidity and regional health disparities. Previously, CEPAL noted that between 2000 and 2015, the years of life without good health in Colombia increased from 5.2 to 5.9 years [23]. This research calculated a DE of 7.4 years for 2018, confirming an increase of 2.2 and 1.5 years, respectively.

The data also reveal that although women have a higher LE than men, these additional years come with more disability, especially in rural areas [18,36,37]. This finding underscores the urgent need to address gender and territorial inequalities in public health policies, with a special focus on rural women.

We require strategies such as a national program to strengthen primary care in these areas, including the construction of clinics, modernization of equipment, and training in geriatrics. Additionally, it is essential to implement mobile clinics that conduct regular visits and awareness campaigns on preventive care in old age [38]. Equally crucial is the need to increase funding and offer tax incentives and bonuses to attract professionals to rural health centers [39].

The negative associations between DFLE and morbidity and disability variables indicate that the burden of disease negatively impacts the quality of life of older adults, highlighting the need to improve both access to and the quality of healthcare services in rural areas [40]. This represents a significant challenge for public health policymakers, who should focus on specific interventions to manage chronic conditions in aging populations, particularly in rural regions [9].

To address this challenge, implementing a National Comprehensive Disability Care Program could help, including early detection, treatment, and rehabilitation for older adults, along with specialized mental health services, thanks to the frequent coexistence of physical disabilities and mental health issues. As an integral part of this program, to substantially improve the quality of life for these individuals, it is essential to create community rehabilitation centers and the training of multidisciplinary teams in rural areas [41,42].

The negative correlations between DFLE and educational variables, such as illiteracy and school absenteeism, emphasize the importance of strengthening lifelong education [7,8,43,44,45]. Evidence shows that higher educational levels relate to greater DFLE, suggesting that policies should include strategies to improve education at all levels and ages [46].

In this context, establishing literacy and continuing education programs for older adults, particularly in rural areas, are useful for improving health and well-being in old age [47]. Implementing these programs through local schools or via distance education platforms using mobile technology is useful for reaching the most remote areas. Additionally, offering incentives such as scholarships and subsidies could encourage the participation of older adults in educational programs [48]. Alongside these, intergenerational initiatives that promote lifelong learning and community support would also be beneficial [49,50].

A relevant finding is the positive relationship between retirement benefits and DFLE, highlighting the importance of income and economic security in the well-being of older adults [16,19]. Policies that ensure adequate pensions and economic support for the elderly can have a significant impact on their quality of life. The state should ensure the implementation of a Universal Pension Program that guarantees a minimum living income for all older adults, especially those in economically vulnerable situations. A national pension fund could sustain this program and supplement it with subsidies for essential expenses, such as medicine and food [51,52]. Additionally, it is a good idea to implement retirement planning programs that allow for the regularization of overdue or delinquent pension contributions, along with strategies for preparing for post-work life [53,54].

The analysis of differences between urban and rural areas in the MFA highlighted the crucial role that area of residence plays in achieving healthy aging. Urban areas had indicators that favor healthy aging compared to rural areas, which, conversely, showed that addressing indicators early in life can help overcome challenges such as poverty, disability, and lack of healthcare at older ages.

The differences in the distribution of the population by gender and age between urban and rural areas of Colombia are crucial for the design of social and health policies, as each region has distinct needs.

In rural areas, it is essential to implement programs that address poverty, improve healthcare, and reduce disability from early ages. A key strategy would be the creation of a Rural Telemedicine Program to enhance access to specialized care in remote communities through the establishment of centers connected to regional hospitals. Private companies can support this program incentivized with tax benefits. Additionally, the study proposes the formation of community managers to facilitate the elderly population’s access to these services, along with offering subsidies and microloans conditioned on the use of telemedicine. Furthermore, training with a focus on elder care and cultural sensitivity is necessary for healthcare personnel to ensure equitable and high-quality access to healthcare services [39,52,55].

In contrast, in urban areas, it is important to continue improving socioeconomic status and educational levels to increase healthy life expectancy. Enhancing public transportation is essential, including exclusive routes for the elderly and people with disabilities, offering regular schedules and adapted vehicles that ensure comfort and accessibility to facilitate mobility and access to essential services [56,57]. Furthermore, promoting affordable and adapted housing is necessary, along with creating communities specifically designed for older adults to foster an inclusive and accessible environment for aging in place [58,59].

It is also important to note that, although urban areas have supported better DFLE outcomes, recent migration influenced some departments characterized as economic and employment centers, particularly the arrival of 2,894,593 individuals in Colombia, making it the main host country for Venezuelan refugees and migrants [60]. This migratory flow, while presenting a challenge to infrastructure and public resources, also offers an opportunity to stimulate the local economy and contribute positively to the country’s fiscal development [60]. However, the scale of the challenge underscores the need for adaptive policies that consider these migratory dynamics in healthcare planning, such as programs aimed at strengthening or expanding mobile health centers that offer free services, potentially managed and funded by international agencies. Additionally, it is important to adopt a life-course approach to the integration of this population, ensuring that regularization and employment efforts are sustainable and equitable, thereby maximizing their contribution to Colombia’s economy and society.

This research has several strengths. The findings are nationally representative and relevant, and they enhance our understanding of the distribution of healthy life expectancy by department and area of residence, supporting the theory that health inequalities, the expansion of morbidity, and well-being are intrinsically linked to social and economic development [4,61]. Implementing policies based on these findings must be approached carefully to develop specific and effective interventions to improve the quality of life of the elderly population, especially in rural areas and among women.

Despite these strengths, this research has some limitations. First, using cross-sectional and self-reported data may affect the reliability of the findings. Cross-sectional data, being a snapshot of a specific moment, limits the ability to establish causal relationships between the studied variables [62,63]. Additionally, self-reported data can introduce reporting biases, which is especially true in older populations who may struggle to recall and accurately report past events [64,65].

Second, there are limitations regarding the omission error that DANE reported in the 2018 National Population and Housing Census. The total omission rate of 8.5% affects the data’s representativeness. Among the departments where this error was evident is the Archipelago of San Andrés and Providencia, where the total omission was 21.2%, which could significantly bias the results for this region [35].

Third, a possible source of measurement bias relates to reporting information for older adults. When another household member provides data, there may be a discrepancy between the perception of health status, disability, and reality, introducing inaccuracies in the data. This situation is common in studies relying on self-reporting, where the accuracy of reports can vary considerably [64,65].

Lastly, regarding the statistical methods used, although MFA is a powerful tool for identifying underlying patterns and relationships among multiple variables, its complexity can make interpretation difficult for those unfamiliar with advanced statistical techniques [66]. Simplifying the explanation of these methods is essential to ensure that all readers understand the process of reaching conclusions. The analysis employed MFA to identify underlying values reflecting the interdependence of variables and to explain most of the total variability with the fewest possible components. This methodology differentiated variables by area of residence, highlighting disparities between urban and rural areas [67].

Ultimately, this study makes a significant contribution to the field of healthy aging by providing empirical evidence on the social inequalities that affect life expectancy in Colombia. The findings add to the existing literature by highlighting how socioeconomic and geographic factors influence the health of the elderly population, offering a solid foundation for future research and public policies aimed at reducing these disparities. It will, therefore, be essential to address the limitations in future studies to improve the precision and applicability of the results. This includes implementing longitudinal studies that can provide a more detailed and precise view of trends in healthy aging and using data collection methods that minimize reporting bias and improve sample representativeness.

## 5. Conclusions

The current Colombian population is undergoing a demographic transition, with decreases in both mortality and birth rates, leading to accelerated aging and a reduction in population growth. As demonstrated in this study, various factors influence the achievement of healthy life years, making it crucial to understand the disparities in aging based on geographic location and economic development. This understanding will enable the development of effective social policies that promote education, economic security, and access to quality health services, with a special focus on women and rural populations.

Finally, it will be essential to ensure access to quality healthcare services, with an emphasis on geriatric care and rural areas. This will require strengthening the training of healthcare professionals and social workers so they can adequately and respectfully address the needs of older adults, promoting awareness and continuous education.

## Figures and Tables

**Table 1:** Processing of study variables.

Social Conditions	Indicator	Operational Definition
*Sense Morbidity* *and Disability*	% Presence of health problems in the last 30 days	$\frac{Pob.60+\;that\;presented\;or\;did\;not\;present\;health\;problems}{Pob.\;total\;60+}\times 100$
	% Physical limitations	$\frac{Pob.60+\;that\;indicated\;having\;or\;not\;having\;a\;PL}{Pob.\;total\;60+}\times 100$
	Disability rate	$\frac{Pob.60+\;that\;reported\;an\;PL}{Pob.\;total\;60+}\times 1000$
	Disability Expectancy (DE) at birth and age 60	(DFLE−LE); (DFLE60−LE60)
*Health Service* *Delivery*	% Attention to health problems	$\frac{Pob.60+\;who\;attended\;a\;health\;institution\;and\;were\;not\;attended}{Pob.\;total\;60+who\;attended\;a\;health\;institution}$ × 100
	% Quality of care	$\frac{Pob.60+\;perception\;of\;quality\;of\;care}{Pob.\;total\;60+that\;was\;treated}\times 100$
*Mortality*	Specific mortality rate (60 years and over)	$\frac{Deaths\;Pob.60\;y+}{Pob.\;total\;60+}\times 1000$
*Healthy Life Expectancy*	DFLE at birth and age 60	$DFLE_{x}=\frac{\sum_{i=x}^{90}\left((1-t_{i})L_{i}\right)}{l_{x}}$
*Educational Status and Capacities*	% People according to literacy	$\frac{Pob.60+\;that\;knows\;or\;does\;not\;read\;and\;write}{Pob.\;total\;60+}\times 100$
	% Attendance at an educational center	$\frac{Pob.60+\;that\;attending\;or\;not\;attending\;an\;EC}{Pob.\;total\;60+}\times 100$
	% Highest educational attainment	$\frac{Pob.60+\;by\;educational\;level\;reached}{Pob.\;total\;60+}\times 100$
*Economic* *conditions*	% Of activities according to work situation	$\frac{Pob.60+\;by\;work\;activity}{Pob.\;total\;60+}\times 100$
	Multidimensional poverty index	$\frac{Pob.60+\;with\;deprivation}{Pob.\;total\;60+}\times 100$

Note: Own elaboration.

**Table 2:** Summary of test statistics and significance.

**Statistic (*W*)**	**N**	**Minimum**	**Maximum**	**Mean**
	68	0.350	0.996	0.890
**Significance (*p*-value)**	68	0.000	1.000	0.310

Note: Own elaboration.

**Table 3:** Indicator groups and identification labels.

Group	Indicators	Label Urban Area	Label Rural Area
*G1. Education*	% Older People in: Illiteracy	AC	AR
	% Old People Level of Studies: Academic Average	MAC	MAR
	% Old People Level of Studies: Technologist	TgC	TgR
	% Old People Level of Studies: University	UC	UR
	% Older People Who Do Not Attend an Educational Center	IC	IR
*G2. Socio-economic*	Multidimensional Poverty Index of Older People	IPM60C	IPM60R
	% Older People Living on Retirement	VJC	VJR
*G3. Sense Morbidity and Disability*	% Older People Who Were Sick	EC	ER
	% Older People with Physical Limitations	LFC	LFR
	Disability Rate	TDC	TDR
	Disability Expectation at Birth	EDC	EDR
	Disability Expectation > 60 years	ED60C	ED60R
*G4. Mortality*	Specific Mortality Rate of Older People	TEMC	TEMR
*G5. Health Care*	% Older People Who Did Not Receive Health Care	NAtnC	NAtnR
	% Older People who Reported Very Poor Quality of Health Care	CMMC	CMMR
*G6. DFLEN*	Disability-Free Life Expectancy at Birth	EVLDC	EVLDR
*G7. DFLE60*	Disability-Free Life Expectancy > 60 years	EVLD60C	EVLD60R

Note: Own elaboration.

**Table 4:** DFLE and DE by sex and area in Colombia 2018.

National	Men	Woman
***LE at Birth***	73.3	79.8
*LE Urban*	74.2	80.3
*LE Rural*	71.2	78.2
***DFLE***	66.9	71.5
*DFLE Urban*	67.7	72.1
*DFLE Rural*	63.1	67.7
***DE***	6.4	8.3
*DE Urban*	6.4	8.2
*DE Rural*	8.1	10.4

Note: Own elaboration from NCPH, 2018.

**Table 5:** Descriptive statistics of LE and DFLE by sex in Colombia 2018.

National	Rango	Mínimum	Máximum	Average	Deviation
*LE at Birth Men*	16.6	60.3	77.0	71.6	3.6935
*LE at Birth Women*	15.1	66.9	82.0	78.1	3.3059
*DFLE Men*	16.4	54.4	70.8	65.6	3.5711
*DFLE Women*	17.1	60.5	77.6	70.3	3.1989

Note: Own elaboration from NCPH, 2018.

**Table 6:** LE60, DFLE60, and DE60 by sex in Colombia 2018.

National	Men	Women
***LE60***	21.1	24.1
*LE60 Urban*	21.3	24.2
*LE60 Rural*	20.6	23.6
***DFLE60***	16.4	18.0
*DFLE60 Urban*	16.7	18.4
*DFLE60 Rural*	14.5	16.0
***DE60***	4.6	6.1
*DE60 Urban*	4.6	5.9
*DE60 Rural*	6.0	7.6

Note: Own elaboration from NCPH, 2018.

**Table 7:** Summary of strong correlations in both sexes, Colombia 2018.

Social Conditions	Variables	Pearson Coefficient	
		DFLE Men	DFLE Women
*Sense Morbidity and Disability*	Sick Yes	−0.41	−0.41
	Physical Limitation Yes	−0.48	−0.49
	Disability Rate	−0.34	−0.53
*Mortality*	Mortality Rate 60 years	0.39	0.35
*Provision of services*	He Was Treated No	−0.72	−0.60
	Very Good Quality	0.36	0.54
	Very Bad Quality	−0.33	−0.12
*Educational situation*	Alphabet No	−0.40	−0.65
	Attendance No	−0.45	0.55
	Preschool	−0.52	−0.48
	Academic or Classical Media	0.61	0.74
	Professional or Technological Technique	0.52	0.70
	University	0.59	0.62
	None	−0.31	−0.58
*Socioeconomic situation*	Lived on retirement, pension, or rent	0.48	0.62
	MPI 60 years	−0.67	−0.64

Note: Own elaboration from NCPH, 2018.

**Table 8:** Association between indicator groups—urban area, Colombia 2018.

Groups	Educational	Socioeconomic	Sense Morbidity	Mortality	Health Care	DFLE	DFLE60
*Educational*	1	0.74	0.34	0.09	0.20	0.34	0.19
*Socioeconomic*	0.74	1	0.20	0.05	0.26	0.22	0.08
*Sense Morbidity*	0.34	0.20	1	0.06	0.14	0.48	0.53
*Mortality*	0.09	0.05	0.06	1	0.12	0.26	0.33
*Healthcare*	0.20	0.26	0.14	0.12	1	0.17	0.13
*DFLE*	0.34	0.22	0.48	0.26	0.17	1	0.80
*DFLE60*	0.19	0.08	0.53	0.33	0.13	0.80	1
***MFA***	**0.67**	**0.58**	**0.64**	**0.46**	**0.48**	**0.75**	**0.71**

Note: Own elaboration.

**Table 9:** Association of indicator groups—rural area, Colombia 2018.

Groups	Educational	Socioeconomic	Sense Morbidity	Mortality	Health Care	DFLE	DFLE60
*Educational*	1	0.81	0.36	0.08	0.25	0.29	0.07
*Socioeconomic*	0.81	1	0.21	0.15	0.22	0.3	0.04
*Sense Morbidity*	0.36	0.21	1	0.22	0.17	0.34	0.52
*Mortality*	0.08	0.15	0.22	1	0.04	0.04	0.09
*Healthcare*	0.25	0.22	0.17	0.04	1	0.33	0.09
*DFLE*	0.29	0.3	0.34	0.04	0.33	1	0.33
*DFLE60*	0.07	0.04	0.52	0.09	0.09	0.33	1
*MFA*	0.71	0.67	0.67	0.38	0.54	0.63	0.49

Note: Own elaboration.

## Data Availability

The data supporting the reported results are available through publicly archived datasets from the Departamento Administrativo Nacional de Estadística (DANE). These include the Censo Nacional de Población y Vivienda 2018 (DANE, 2018a), accessible at https://www.dane.gov.co/index.php/estadisticas-por-tema/demografia-y-poblacion/censo-nacional-de-poblacion-y-vivenda-2018/cuantos-somos (accessed on 22 September 2023); the Cuestionario del Censo Nacional de Población y Vivienda 2018 (DANE, 2018b) at https://www.dane.gov.co/files/censo2018/informacion-tecnica/Cuestionario_Hogares.pdf (accessed on 22 September 2023); and the REDATAM Censo Nacional de Población y Vivienda 2018 (DANE, 2018d) at http://systema59.dane.gov.co/bincol/RpWebEngine.exe/Portal?BASE=CNPVBASE4V2&lang=esp (accessed on 22 September 2023).
